# Influence of Adult Attachment on COVID-19 Vaccination Intention: The Mediating Roles of Help-Seeking Style and Professional Help-Seeking Behavior

**DOI:** 10.3390/vaccines10020221

**Published:** 2022-01-30

**Authors:** Junyu Lu, Runzan Zhang, Xinping Zhang

**Affiliations:** 1 School of Medicine and Health Management, Tongji Medical College, Huazhong University of Science and Technology, Wuhan 430030, China; junyu_lu12@hust.edu.cn; 2 Tongji School of Pharmacy, Huazhong University of Science and Technology, Wuhan 430030, China; zrz_54@hust.edu.cn

**Keywords:** COVID-19 vaccination intention, adult attachment, help-seeking style, professional help-seeking, mediation role

## Abstract

Vaccination against COVID-19 is regarded as one of the most promising interventions to control the pandemic. This study aimed to examine whether adult attachment affects an individual’s COVID-19 vaccination intention and whether this relationship is mediated by help-seeking style and professional help-seeking behavior. A total of 401 Chinese adults participated in this online cross-sectional survey. The questionnaires for adult attachment (Depend, Close, and Anxiety), help-seeking style (dependency, autonomy, and avoidance), professional help-seeking behavior, and COVID-19 vaccination intention were rated on five-point or seven-point Likert scales, with satisfactory reliability (Cronbach’s α values were all >0.80). Structural equation modelling was used to construct path models based on the above elements. Higher scores in the Depend (Effect = 0.047, SE = 0.018, 95% CI = [0.019, 0.093]) and Close dimensions of attachment (Effect = 0.028, SE = 0.014, 95% CI = [0.007, 0.065]) predicted a stronger dependency-oriented help-seeking style, which thus predicted greater vaccination intention. Higher scores in the Close dimension (Effect = 0.007, SE = 0.004, 95% CI = [0.001, 0.018]) and lower scores in the Anxiety dimension of attachment (Effect = −0.003, SE = 0.002, 95% CI = [−0.008, −0.001]) predicted a stronger autonomy-oriented help-seeking style and further predicted more professional help-seeking behaviors, which promoted greater COVID-19 vaccination intention. The results of this study indicate that help-seeking moderates the relationship between adult attachment and COVID-19 vaccination intention. Guiding help-seeking behavior for individuals with different attachment styles may be an entry point for improving COVID-19 vaccination intention.

## 1. Introduction

COVID-19 has quickly spread across the globe, infecting more than 197 million and killing more than 4.2 million as of 26 July 2021 [1]. Vaccination against COVID-19 has been regarded as one of the most promising health interventions to prevent and control the pandemic, significantly reducing the number of patients with severe cases and the mortality rate [2], and contributing to protecting the population infected with the B.1.617.2 (delta) variant [3]. Despite the availability of COVID-19 vaccination services, there has been a delay in public acceptance and refusal of the vaccination, which is known as vaccine hesitancy [4]. Vaccine hesitancy was identified by the World Health Organization (WHO) as one of the top ten threats to global health in 2019, hindering the construction of herd immunity, global health, and economic recovery [5,6].

A global survey of the potential acceptance of a COVID-19 vaccine reported that most countries had a high COVID-19 burden [7]. The proportion of respondents with intention of accepting immediate COVID-19 vaccination was 23% in China [2], and 71.5% of respondents indicated that safety and effectiveness were important factors for COVID-19 vaccination [7]. In addition, risk perception, vaccination convenience, and vaccination history also affected COVID-19 vaccination intention [6,8]. However, individual psychological processes and people’s recommendations were found to be important factors affecting COVID-19 vaccination intention and further study is needed in this area [2,9,10].

### 1.1. Adult Attachment and COVID-19 Vaccination Intention

Attachment is formed in the earliest stages of life and promotes infants’ sense of security and survival ability by forming emotional bonds with their caregivers [11]. Adult attachment is the continuation and reappearance of early attachment experience, which refers to the tendency of individuals to form an emotional connection with others [12]. When people encounter stressful events, attachment is activated, eliciting individuals’ expectations, perceptions, and behavior [13]. Scholars describe three main dimensions of adult attachment: Depend (the extent to which an individual feels he or she can depend on others), Close (the extent to which an individual is comfortable with closeness), and Anxiety (the extent to which an individual is anxious or fearful about things such as being abandoned or unloved) [12]. Attachment avoidance refers to low levels of Depend and Close [14]. Adult attachment, as a long-term and stable personality trait, affects coping styles and health behaviors, has become increasingly important in psychosomatic researches [15]. Previous studies have demonstrated that high attachment avoidance (low Depend or Close) or Anxiety of attachment in individuals led to more frequent health risk behaviors, especially substance use and treatment nonadherence [16,17]. In contrast, individuals with low attachment avoidance demonstrated higher treatment compliance. Furthermore, adult attachment was found to influence compliance with health guidelines [18]. In 2021, researchers suggested that under the stress of COVID-19, individuals with high Anxiety of attachment were less likely to adhere to government-recommended health guidelines regarding COVID-19 [9]. Therefore, Hypothesis 1 is proposed in this study:

**Hypothesis** **1** **(H1).** *Adult attachment contributes to COVID-19 vaccination intention.*

**Hypothesis** **1a** **(H1a).** *The Depend and Close dimensions of attachment positively influence COVID-19 vaccination intention.*

**Hypothesis** **1b** **(H1b).** *The Anxiety dimension of attachment negatively influences COVID-19 vaccination intention.*

### 1.2. Mediators of Adult Attachment and COVID-19 Vaccination Intention: Help-Seeking Style and Professional Help-Seeking

The above studies suggest some potential associations between adult attachment and COVID-19 vaccination intention. However, this relationship was mostly gleaned from indirect, empirical evidence. It is necessary to identify the path of the effect of adult attachment on COVID-19 vaccination intention during the period of the COVID-19 global epidemic and to examine potential mediators between the two elements. Previous studies have indicated that adult attachment could affect individual help-seeking behavior [19,20]. Additionally, the impact of help-seeking on vaccination behavior has also been demonstrated [21]. Thus, help-seeking seems to be a potential mediator of the relationship between adult attachment and COVID-19 vaccination intention.

Individual help-seeking styles are relatively stable and include dependency-oriented (asking someone else to fix a problem), autonomy-oriented (asking for help to learn how to fix a problem), and avoidance-oriented (not seeking any assistance and coping on their own) help-seeking, which differ from problem-oriented actual help-seeking behavior [20]. Dependency-oriented help-seeking leaves them dependent on assistance the next time they experience a similar difficulty. Conversely, autonomy-oriented help-seeking allows individuals to be self-reliant the next time they encounter the same problem. Regarding the relationship between adult attachment and help-seeking style, scholars found that individuals with high Depend traits expressed stable personal preferences for dependency-oriented help, which led to a readiness to be fully reliant on others. Individuals with an autonomy-oriented help-seeking style had approach (Close) temperaments and invested more efforts to solve the problem at hand and in turn were better equipped with tools to solve similar problems in the future. Meanwhile, avoidant individuals expressed an avoidance-oriented help-seeking style and refrained from help-seeking even at high stress levels [19,20]. The effect of help-seeking style on health behavior decision-making has been confirmed by previous studies. Because dependent people were hypothesized to feel helpless and in need of guidance and support from others, they demonstrated an elevated rate of help-seeking behaviors in a variety of settings and their decision-making behavior was consistent with that of the help-seekers [22]. In contrast, avoidance-oriented help-seekers with low dependence emphasized self-independence, and their decision-making behavior was associated with the perception of personal goal achievement and motivation [23]. Since studies have claimed that more help-seeking behavior is associated with higher vaccine acceptance [21], dependency-oriented and autonomy-oriented help-seeking styles may predict greater COVID-19 vaccination intention, while avoidance-oriented help-seeking style may predict lower intention. Therefore, Hypothesis 2 is proposed in this study:

**Hypothesis** **2** **(H2).** *Help-seeking style mediates the association between adult attachment and COVID-19 vaccination intention.*

**Hypothesis** **2a** **(H2a).** *The Depend dimension of attachment positively influences dependency-oriented and autonomy-oriented help-seeking styles, while it negatively influences the avoidance-oriented help-seeking style.*

**Hypothesis** **2b** **(H2b).** *The Close dimension of attachment positively influences dependency-oriented and autonomy-oriented help-seeking styles, while it negatively influences the avoidance-oriented help-seeking style.*

**Hypothesis** **2c** **(H2c).** *The Anxiety dimension of attachment negatively influences dependency-oriented and autonomy-oriented help-seeking styles, while it positively influences avoidance-oriented help-seeking style.*

**Hypothesis** **2d** **(H2d).** *Dependency-oriented help-seeking style positively influences COVID-19 vaccination intention.*

**Hypothesis** **2e** **(H2e).** *Autonomy-oriented help-seeking style positively influences COVID-19 vaccination intention.*

**Hypothesis** **2f** **(H2f).** *Avoidance-oriented help-seeking style negatively influences COVID-19 vaccination intention.*

Regarding the relationship between adult attachment and professional help-seeking behavior, researchers have suggested that individuals who were aware of help-seeking by others that were close to them were two times more likely to seek professional help [24]. Meanwhile, the link between professional help-seeking and COVID-19 vaccination intention has been demonstrated in some studies. Seeking help from professionals such as doctors could improve individuals’ intention to get vaccinated under the stress of the COVID-19 outbreak [7,8,9]. One study demonstrated that asking professional colleagues for help was a desirable social norm that was able to increase the coverage rate of the COVID-19 vaccine [21]. Therefore, Hypothesis 3 is proposed in this study:

**Hypothesis** **3** **(H3).** *Professional help-seeking behavior mediates the association between adult attachment and COVID-19 vaccination intention.*

**Hypothesis** **3a** **(H3a).** *The Close dimension of attachment positively influences professional help-seeking behavior.*

**Hypothesis** **3b** **(H3b).** *Professional help-seeking behavior positively influences COVID-19 vaccination intention.*

Importantly, help-seeking style can predict professional help-seeking behavior. Researchers found that autonomy-oriented help-seeking (aimed to learn skills to fix a problem) allowed individuals to be self-reliant the next time they encountered a similar problem, which might predict more professional help-seeking preferences [20]. Furthermore, these individuals often turned to authorities or experts [25,26]. Thus, it can be inferred that help-seeking style may influence one’s professional help-seeking behavior. Therefore, Hypothesis 4 is proposed in this study:

**Hypothesis** **4** **(H4).** *Help-seeking style and professional help-seeking behavior together play a serial mediating role in the association between adult attachment and COVID-19 vaccination intention.*

**Hypothesis** **4a** **(H4a).** *Autonomy-oriented help-seeking style positively influences professional help-seeking behavior.*

**Figure 1:**
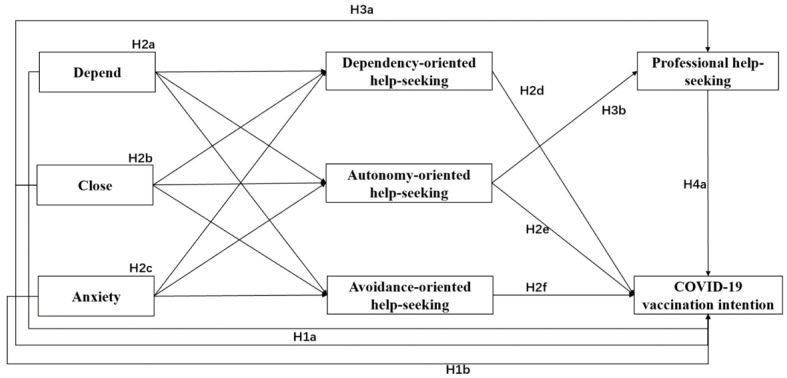
Hypothesized pathways from adult attachment to COVID-19 vaccination intention in the measurement model.

The aim of this study was to investigate the relationship between adult attachment and COVID-19 vaccination intention, and to explore the mediating role of help-seeking style and professional help-seeking behavior. Figure 1 presents the measurement model in this study.

## 2. Methods

### 2.1. Participants and Procedure

The present online questionnaire survey was conducted from 3 August 2021 to 11 August 2021 using non-probability convenience sampling to select samples in Zhejiang Province. The sample size was estimated using the calculation formula of a cross-sectional study design: *N* = $Z_{1-\alpha /2}^{2}$ p(1 − p)/d^2^ (α = 0.05, $Z_{1-\alpha /2}$ = 1.96, d = 0.05, p = 30%, p refers to the rate that people do not seek help in the pilot investigation) [27]. Inclusion criteria were being able to read and understand the questionnaire, living in China, and being at least 18 years old. The exclusion criteria were submitting inconsistent responses, such as participants scoring high on both dependency-oriented and avoidance-oriented help-seeking styles. Fully informed consent was obtained from the participants in this survey.

A total of 401 participants effectively completed the questionnaires independently, which met the required sample size (*N* ≥ 323), of whom 62.59% were females (*N* = 251). The average age was 33.16 with a standard deviation of 11.73. Overall, 71.57% of participants had a bachelor’s degree or higher qualification, and 63.84% had an annual household income ranging from CNY 50,000 to CNY 150,000 (USD 7730 to 23,190). In total, 21.95% studied medical-related majors, 9.73% were staff of relevant government departments or teachers, and 45.14% were workers. During the survey, 41.90% of the participants indicated that there had been a confirmed or suspected COVID-19 case in their county (area), and 19.70% reported that they refused vaccination.

### 2.2. Measures

#### 2.2.1. Adult Attachment

The Adult Attachment Scale (AAS, 18 items) was applied to assess continuous adult attachment dimensions and to analyze individual attachment phenomena accurately [12]. The AAS consisted of three dimensions: Depend, Close, and Anxiety. Depend contained six items regarding the extent to which individuals felt they could depend on others, such as “I am comfortable depending on others”. Close contained six items regarding the extent to which an individual was comfortable with closeness, such as “I find it relatively easy to get close to others”. Anxiety contained six items regarding the extent to which an individual was anxious or fearful about things such as being abandoned or unloved, for example, “I find others are reluctant to get as close as I would like”. Satisfactory Cronbach’s α values for three dimensions were reported (“Depend”, 0.819; “Close”, 0.868; and “Anxiety”, 0.934). Each item was rated on a seven-point Likert scale, ranging from 1 (not at all true of me) to 7 (very true of me). Higher scores on these items of Depend and Close indicated lower attachment avoidance, whereas higher scores on Anxiety indicated higher attachment anxiety.

#### 2.2.2. Help-Seeking Style

The Help-Seeking Style Scale (14 items) was used to measure individuals’ relatively stable help-seeking preferences [20,28]. It consisted of three dimensions: (a) Dependency-oriented help-seeking contained five items, such as “I frequently ask for help with a problem before I try to solve it on my own”. (b) Autonomy-oriented help-seeking contained four items, such as “When I encounter a problem, I talk to someone to improve my ability to cope with it”. (c) Avoidance-oriented help-seeking contained five items, such as “When I try to solve a problem, I count on myself alone and not on anyone else”. Satisfactory Cronbach’s α values for three dimensions were reported (“Dependency”, 0.817; (2) “Autonomy”, 0.869; and “Avoidance”, 0.872). Each item was rated on a seven-point Likert scale, ranging from 1 (not at all true of me) to 7 (very true of me). A higher score of a dimension indicated a stronger corresponding help-seeking style.

#### 2.2.3. Professional Help-Seeking Behavior

The General Help-seeking Questionnaire (GHSQ) was used to measure professional help-seeking behavior [29]. The section on professional help-seeking (e.g., a physician) was adopted, defining the applicable condition as “The likelihood that you will seek help from the following prior to your COVID-19 vaccination”, which were rated on a seven-point Likert scale, ranging from 1 (extremely unlikely) to 7 (extremely likely) to assess an individual’s likelihood of seeking help from a professional source or past help-seeking experiences.

#### 2.2.4. COVID-19 Vaccination Intention

In the present study, one item represented the degree of participants’ intention to take the COVID-19 vaccine, and was rated on a five-point Likert scale from 1 (completely disagree) to 5 (completely agree). A higher score presented a higher COVID-19 vaccination intention.

### 2.3. Data Analysis

SPSS 24.0 (IBM SPSS, Chicago, IL, USA) was used to clean data and conduct correlation analysis. The *t*-test and one-way ANOVA were used to test the differences in COVID-19 vaccination intention in different sociodemographic characteristics, and multiple linear regression was used to further screen the variables that were statistically significant in the univariate analysis. Finally, age was included as a covariate in the mediation model. Bivariate correlations between adult attachment, help-seeking style, professional help-seeking behavior, and COVID-19 vaccination intention were assessed. Pearson correlation was used for continuous variables, and Spearman correlation was used for ordinal variables. Structural equation modelling was done to test the direct and indirect pathways from adult attachment (Depend, Close, and Anxiety) to COVID-19 vaccination intention, including two stages: Validation of the measurement model and fitting of the structural models, using the maximum-likelihood method in AMOS 24.0 software (IBM SPSS, Chicago, IL, USA). According to Hoyle and Panter [30], several fit indices were used including χ^2^/df (values of 3 or less), the comparative fit index (CFI, values of 0.90 or greater), the root mean square error of approximation (RMSEA, values of 0.08 or less), and the standardized root-mean-square residual (SRMR, values of 0.08 or less) to assess goodness of fit for models. The average of each item was used in the model. All mediations were tested with 2000 bootstrapped iterations, and standardized coefficients were calculated. Significance was considered at 95% bias-corrected confidence intervals not including 0 or 2-sided *p* < 0.05.

## 3. Results

### 3.1. Correlation Analysis

The means, standard deviations (SDs), and correlations are shown in Table 1. The results of the correlation analysis indicated that the correlation between each pair of variables was significant among adult attachment, help-seeking behavior, professional help-seeking behavior, and COVID-19 vaccination intention. Notably, score in the Depend dimension (r = 0.134, *p* < 0.01), dependency-oriented, and autonomy-oriented help-seeking style (r = 0.102, *p* < 0.05; r = 0.227, *p* < 0.001) were all positively associated with professional help-seeking behavior, and all three of the above were in turn positively correlated with COVID-19 vaccination intention (all *p* < 0.05). Furthermore, the autonomy-oriented help-seeking style was positively correlated with professional help-seeking behavior and COVID-19 vaccination intention (all *p* < 0.05).

### 3.2. Mediating Model Analysis

**Figure 2:**
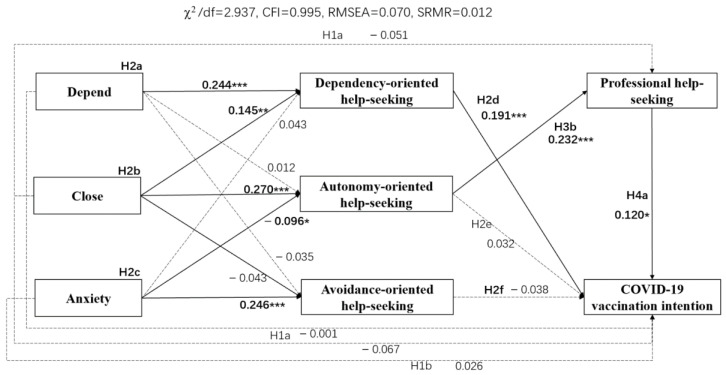
Mediated model 1. *N* = 401. All coefficients are standardized regression weights. Dotted arrows refer to nonsignificant effects. * *p* < 0.05. ** *p* < 0.01. *** *p* < 0.001.

Figure 2 shows the coefficients between the independent variable (adult attachment), mediating variables (help-seeking style and professional help-seeking behavior), and the outcome variable (COVID-19 vaccination intention) as well as the model fitting.

The results in Table 2 indicate that higher scores in the Depend (Effect = 0.047, SE = 0.018, 95% CI = [0.019, 0.093]) and Close attachment dimensions (Effect = 0.028, SE = 0.014, 95% CI = [0.007, 0.065]) were associated with stronger predicted dependency-oriented help-seeking style, and thus greater predicted vaccination intention. Additionally, higher scores in the Close dimension (Effect = 0.007, SE = 0.004, 95% CI = [0.001, 0.018]) and lower scores in the Anxiety dimension of attachment (Effect = −0.003, SE = 0.002, 95% CI = [−0.008, −0.001]) were associated with a stronger predicted autonomy-oriented help-seeking style, which further predicted more professional help-seeking behaviors and promoted greater COVID-19 vaccination intention. However, we found no evidence that adult attachment directly affected COVID-19 vaccination intention, nor did we find a direct effect of Close dimension on professional help-seeking behavior. Therefore, help-seeking style and professional help-seeking behavior completely mediated the relationship between adult attachment and COVID-19 vaccination intention, since the effects of independent on dependent variable were not significant.

## 4. Discussion

### 4.1. Main Findings of This Study

This study established that help-seeking style and professional help-seeking behavior completely mediated the relationship between adult attachment and COVID-19 vaccination intention. Higher scores in the Depend and Close dimensions of attachment indirectly predicted greater COVID-19 vaccination intention through a stronger dependency-oriented help-seeking style. Higher scores in the Close dimension and lower scores in the Anxiety dimension of attachment indirectly predicted greater COVID-19 vaccination intention through a serial mediation of stronger autonomy-oriented help-seeking style and professional help-seeking behavior.

### 4.2. Comparison with Existing Literature

Our results suggest a tight connection between adult attachment and dependency-oriented help-seeking style, and between dependency-oriented help-seeking style and COVID-19 vaccination intention. Participants demonstrated a stronger dependency-oriented help-seeking style if they tended to depend on others or were comfortable with closeness, and this predicted a higher intention to get vaccinated against COVID-19. This is consistent with the findings of Komissarouk et al., who concluded that two different kinds of self-construal, dependent and independent, affected the type of help-seeking: Those with stronger dependent traits demonstrated a more stable dependency-oriented help-seeking preference relative to those with independent traits, and dependence positively predicted dependency-oriented help-seeking style [31]. Similar results were found in studies on two-dimensional adult attachment [14], indicating that individuals with lower attachment avoidance rely on help-seeking to solve problems [20]. The reason may be that people with high scores in the Depend and Close dimensions of attachment are more likely to trust others, while individuals with attachment avoidance view others negatively and tend to devalue the importance of others and distance themselves from others to avoid seeking help [32]. Studies have demonstrated that a lack of trust in government was one of the most common reasons for responding “no” regarding vaccination intent [33,34]. A higher level of confidence in the government was associated with a greater intention to get the COVID-19 vaccine and take advice; otherwise, people presented higher odds of refusal (No versus Yes, aOR = 7.56, 95% CI = [3.59, 15.92]; No versus Wait, aOR = 4.54, 95% CI = [2.33, 8.87]) [6,7]. Furthermore, it was found that people from collectivistic cultural areas preferred to trust others, and therefore reported a higher preference for a dependence-oriented help-seeking style than those from individualistic cultural areas [31]. A study observed that the probability of getting vaccinated against the flu was 5.52% higher among healthcare employees in conditions where most colleagues got vaccinated, compared to those in conditions where most colleagues did not have the flu vaccine [21]. Therefore, collectivist cultural areas with high trust in the government have relatively high vaccination rates.

Our results demonstrate that individuals with higher scores in the Close dimension and those with lower scores in the Anxiety dimension of attachment were more likely to form an autonomy-oriented help-seeking style, which was associated with more professional help-seeking behaviors, thus promoting COVID-19 vaccination intention. This result overlapped with findings indicating that individuals who perceived formal help-seeking behavior in those that were close to them were more likely to demonstrate professional help-seeking behavior [24]. The Anxiety dimension of attachment represents an individual’s self-stigma and fear of being rejected. Studies demonstrated that higher a higher score in Anxiety dimension of attachment predicted higher self-denial, which in turn devalued an individual’s self-worth and overvalued the opinions of others that were close to them (family or friends), thereby predicting a stronger intention to rely on others’ support and a weaker intention to seek professional counseling to solve problems [35]. This was consistent with our results, demonstrating that professional help-seeking behavior played a masking effect in the relationship between Anxiety of attachment and decision making.

In addition, individuals’ autonomy-oriented help-seeking style was a proximal predictor of professional help-seeking behavior. Professional help-seeking from this perspective reflects a person’s desire to use help in order to achieve personal development [19]. Previous literature found that help-seeking style could predict patient preferences for being involved in shared medical decision-making. As Barry and Edgman-Levitan suggested, shared decision-making between patients and clinicians was optimal [36], but it was based on two situations: First, for patients with high scores in Depend and Close dimensions of attachment, participation in shared decision-making was related to a strong dependency-oriented help-seeking style because of their full dependence on clinicians and medical information; second, for patients with secure attachment (high in Close and low in Anxiety dimensions) [12], patients had a strong desire for self-improvement, which was associated with an autonomy-oriented help-seeking style, thereby promoting the behavior of seeking help from medical professionals. Therefore, adult attachment affected medical decision-making through the mediating role of dependency-oriented help-seeking, as well as the serial mediating effects of professional help-seeking behavior and autonomy-oriented help-seeking style. Studies on vaccine hesitancy have highlighted that vaccination safety information from authoritative sources and advice from valued healthcare professionals were important to alleviate vaccine hesitancy [4,37], which has also been suggested in research about COVID-19 vaccination acceptance [38].

The evidence in this study is important because it demonstrates how COVID-19 vaccination intention is influenced by adult attachment and reveals the significance of psychological determinants. Public organizations and their managers may help people with different attachment states and help-seeking preferences by implementing intervention programs to improve their ability to adapt to new situations. Social norms may be an effective intervention to promote COVID-19 vaccination coverage, because “people like to do what most people actually do” [39]. Individuals with strong dependency traits are more likely to have consistent vaccination behaviors with the majority of people in their organization due to the preference for a strong dependency-oriented help-seeking style. Emphasizing the social norm that most people get a flu shot may induce more individuals to adopt the same behavior, thus improving COVID-19 vaccine coverage. Additionally, interventions to motivate people with autonomy-oriented help-seeking preferences to achieve a focused goal [40] (perhaps by successfully protecting themselves or others from infection) are of great importance, as they promote professional help-seeking behavior, thereby increasing the probability of COVID-19 vaccination. Furthermore, given that expanding coverage is inseparable from the high recognition of vaccines by medical professionals, it has become very urgent for policy-makers to develop educational programs.

### 4.3. Strengths and Limitations

This study is the first to examine the mediating roles of help-seeking style and professional help-seeking behavior between adult attachment and an individual’s COVID-19 vaccination intention, providing evidence for a new perspective in promoting vaccination. However, several limitations in this study are acknowledged. First, a convenient sampling method was used in this survey in developing regions, but random sampling was not conducted, which may have affected the representativeness of the samples, and it was not determined whether the findings can be generalized to China or other countries. Second, these findings were based on self-reported scales, which implies the possibility of self-report bias. Additionally, some sociodemographic variables were not taken into account in this paper, which may have led to bias. Third, as a cross-sectional survey, causality could not be inferred. This is one of the most important problems to be solved in our follow-up investigation. Further study needs to engage a large representative group of participants in a longitudinal study, to focus on the actual behavior regarding COVID-19 vaccination, and should discuss more potential variables.

## 5. Conclusions

Help-seeking style and professional help-seeking behavior mediated the relationship between adult attachment and COVID-19 vaccination intention. Guiding help-seeking behavior for individuals with a different attachment style may be an entry point for improving COVID-19 vaccination intention.

## Figures and Tables

**Table 1:** Means, standard deviations, and correlations among variables for the measurement model.

Variable	Mean	SD ^a^	1	2	3	4	5	6	7	8
1 Depend	4.110	1.651	1							
2 Anxiety	3.360	1.734	0.257 ***	1						
3 Close	4.620	1.564	0.379 ***	0.055	1					
4 Dependency	4.876	1.731	0.310 ***	0.114 *	0.240 ***	1				
5 Autonomy	5.693	1.261	0.210 ***	−0.033	0.363 ***	0.448 **	1			
6 Avoidance	3.302	1.729	−0.070	0.205 ***	−0.106 *	−0.258 ***	−0.218 ***	1		
7 Professional help–seeking behavior	4.999	1.640	0.134 **	−0.010	0.070	0.102 *	0.227 ***	−0.028	1	
8 COVID-19 vaccination intention	2.025	3.281	0.068 **	0.033 *	0.017 *	0.226 ***	0.152 **	−0.097	0.148 **	1

*N* = 401. ^a^ SD: standard deviation. * *p* < 0.05. ** *p* < 0.01. *** *p* < 0.001.

**Table 2:** Standardized indirect path effects from adult attachment to COVID-19 vaccination intention.

Path	Effect	SE	95% LLCI	95% ULCI
Total indirect effect	0.079	0.025	0.035	0.133
Indirect effect 1: Depend→Dependency-oriented help-seeking→COVID-19 vaccination intention	0.047	0.018	0.019	0.093
Indirect effect 2: Close→Dependency-oriented help-seeking→COVID-19 vaccination intention	0.028	0.014	0.007	0.065
Indirect effect 3: Close→Autonomy-oriented help-seeking→Professional help-seeking→COVID-19 vaccination intention	0.007	0.004	0.001	0.018
Indirect effect 4: Anxiety→Autonomy-oriented help-seeking→Professional help-seeking→COVID-19 vaccination intention	−0.003	0.002	−0.008	−0.001

## Data Availability

The datasets generated during the current study are not publicly available but are available from the corresponding author on reasonable request.

## References

[1] WHO Coronavirus Disease (COVID-19) Dashboard. https://covid19.who.int/.

[2] Wang J.H., Lu X.R., Lai X.Z., Lyu Y., Zhang H.J., Fenghuang Y.F., Jing R.Z., Li L., Yu W.Z., Fang H. (2021). The Changing Acceptance of COVID-19 Vaccination in Different Epidemic Phases in China: A Longitudinal Study. Vaccines.

[3] Bernal J.L., Andrews N., Gower C., Gallagher E., Simmons R., Thelwall S., Stowe J., Tessier E., Groves N., Dabrera G. (2021). Effectiveness of COVID-19 Vaccines against the B.1.617.2 (Delta) Variant. N. Engl. J. Med..

[4] Macdonald N.E. (2015). Vaccine hesitancy: Definition, scope and determinants. Vaccine.

[5] WHO Ten Threats to Global Health in 2019. https://www.who.int/news-room/spotlight/ten-threats-to-global-health-in-2019.

[6] Soares P., Rocha J.V., Moniz M., Gama A., Laires P.A., Pedro A.R., Dias S., Leite A., Nunes C. (2021). Factors Associated with COVID-19 Vaccine Hesitancy. Vaccines.

[7] Lazarus J.V., Ratzan S.C., Palayew A., Gostin L.O., El-Mohandes A. (2021). A global survey of potential acceptance of a COVID-19 vaccine. Nat. Med..

[8] Lin C., Tu P., Beitsch L.M. (2020). Confidence and Receptivity for COVID-19 Vaccines: A Rapid Systematic Review. Vaccines.

[9] Segal S., Sharabany R., Maaravi Y. (2021). Policymakers as safe havens: The relationship between adult attachment style, COVID-19 fear, and regulation compliance. Pers. Individ. Dif..

[10] Wang Y.Y., Zhang X.P. (2021). Influence of Parental Psychological Flexibility on Pediatric COVID-19 Vaccine Hesitancy: Mediating Role of Self-Efficacy and Coping Style. Front. Psychol..

[11] Bowlby J. (1977). The making and breaking of affectional bonds. I. Aetiology and psychopathology in the light of attachment theory. An expanded version of the Fiftieth Maudsley Lecture, delivered before the Royal College of Psychiatrists, 19 November 1976. Br. J. Psychiatry.

[12] Collins N.L., Read S.J. (1990). Adult attachment, working models, and relationship quality in dating couples. J. Pers. Soc. Psychol..

[13] Bowlby J. (1973). Attachment and loss: Volume II: Separation, anxiety and anger. Attachment and Loss: Volume II: Separation, Anxiety and Anger.

[14] Vogel D.L., Wei M.F. (2005). Adult attachment and help-seeking intent: The mediating roles of psychological distress and perceived social support. J. Couns. Psychol..

[15] Ravitz P., Maunder R., Hunter J., Sthankiya B., Lancee W. (2010). Adult attachment measures: A 25-year review. J. Psychosomat. Res..

[16] Maunder R.G., Hunter J.J. (2001). Attachment and psychosomatic medicine: Developmental contributions to stress and disease. Psychosom. Med..

[17] Vaillant G.E. (1974). Natural history of male psychological health. II. Some antecedents of healthy adult adjustment. Arch. Gen. Psychiatry.

[18] Ciechanowski P.S., Katon W.J., Russo J.E., Walker E.A. (2001). The patient-provider relationship: Attachment theory and adherence to treatment in diabetes. Am. J. Psychiatry.

[19] Nadler A. (1997). Personality and help seeking. Sourcebook of Social Support and Personality.

[20] Komissarouk S., Harpaz G., Nadler A. (2017). Dispositional differences in seeking autonomy- or dependency-oriented help: Conceptual development and scale validation. Pers. Individ. Dif..

[21] Belle N., Cantarelli P. (2021). Nudging Public Employees Through Descriptive Social Norms in Healthcare Organizations. Public Adm. Rev..

[22] Bornstein R.F. (1992). The dependent personality: Developmental, social, and clinical perspectives. Psychol. Bull..

[23] Karabenick S.A. (2004). Perceived achievement goal structure and college student help seeking. J. Educ. Psychol..

[24] Disabato D.J., Short J.L., Lameira D.M., Bagley K.D., Wong S.J. (2018). Predicting help-seeking behavior: The impact of knowing someone close who has sought help. J. Am. Coll. Health.

[25] Karabenick S.A., Knapp J.R. (1988). Effects of Computer Privacy on Help-Seeking. J. Appl. Soc. Psychol..

[26] Bamberger P. (2009). Employee help-seeking: Antecedents, consequences and new insights for future research. Res. Pers. Hum. Resour. Manag..

[27] Murti B. (2006). Desain dan ukuran sampel untuk penelitian kuantitatif dan kualitatif di bidang kesehatan. Yogyak. Gadjah Mada Univ. Press.

[28] Lannin D.G., Barrowclough M., Vogel D.L. (2020). An examination of help-seeking preferences via best-worst scaling. J. Clin. Psychol..

[29] Wilson C.J., Deane F.P., Ciarrochi J., Rickwood D. (2005). Measuring Help-Seeking Intentions: Properties of the General Help-Seeking Questionnaire. Can. J. Couns..

[30] Hoyle R., Panter A. (1995). Writing about structural equation models. Structural Equation Modeling: Concepts, Issues, and Applications.

[31] Komissarouk S., Nadler A. (2014). “I” Seek Autonomy, “We” Rely on Each Other: Self-Construal and Regulatory Focus as Determinants of Autonomy- and Dependency-Oriented Help-Seeking Behavior. Pers. Soc. Psychol. Bull..

[32] Mikulincer M., Shaver P.R., Pereg D. (2003). Attachment Theory and Affect Regulation: The Dynamics, Development, and Cognitive Consequences of Attachment-Related Strategies. Motiv. Emot..

[33] Fisher K.A., Bloomstone S.J., Walder J., Crawford S., Fouayzi H., Mazor K.M. (2020). Attitudes toward a Potential SARS-CoV-2 Vaccine a Survey of US Adults. Ann. Intern. Med..

[34] Sallam M., Dababseh D., Eid H., Al-Mahzoum K., Al-Haidar A., Taim D., Yaseen A., Ababneh N.A., Bakri F.G., Mahafzah A. (2021). High Rates of COVID-19 Vaccine Hesitancy and Its Association with Conspiracy Beliefs: A Study in Jordan and Kuwait among Other Arab Countries. Vaccines.

[35] Cheng H.-L., McDermott R.C., Lopez F.G. (2015). Mental Health, Self-Stigma, and Help-Seeking Intentions Among Emerging Adults: An Attachment Perspective. Couns. Psychol..

[36] Barry M.J., Edgman-Levitan S. (2012). Shared decision making—The pinnacle of patient-centered care. N. Engl. J. Med..

[37] Larson H.J., Jarrett C., Eckersberger E., Smith D.M.D., Paterson P. (2014). Understanding vaccine hesitancy around vaccines and vaccination from a global perspective: A systematic review of published literature, 2007–2012. Vaccine.

[38] Wang J.H., Jing R.Z., Lai X.Z., Zhang H.J., Lyu Y., Knoll M.D., Fang H. (2020). Acceptance of COVID-19 Vaccination during the COVID-19 Pandemic in China. Vaccines.

[39] Leonard T.C. (2008). Richard, H. Thaler, Cass, R. Sunstein. Nudge: Improving Decisions about Health, Wealth, and Happiness. Constit. Polit. Econ..

[40] Briley D.A., Aaker J.L. (2006). Bridging the Culture Chasm: Ensuring That Consumers Are Healthy, Wealthy, and Wise. J. Public Policy Mark..

